# Molecular analysis of endothelial progenitor cell (EPC) subtypes reveals two distinct cell populations with different identities

**DOI:** 10.1186/1755-8794-3-18

**Published:** 2010-05-13

**Authors:** Reinhold J Medina, Christina L O'Neill, Mark Sweeney, Jasenka Guduric-Fuchs, Tom A Gardiner, David A Simpson, Alan W Stitt

**Affiliations:** 1 Centre for Vision & Vascular Science, School of Medicine, Dentistry & BioMedical Science, Queen's University Belfast, Belfast, BT12 6BA, UK

## Abstract

**Background:**

The term endothelial progenitor cells (EPCs) is currently used to refer to cell populations which are quite dissimilar in terms of biological properties. This study provides a detailed molecular fingerprint for two EPC subtypes: early EPCs (eEPCs) and outgrowth endothelial cells (OECs).

**Methods:**

Human blood-derived eEPCs and OECs were characterised by using genome-wide transcriptional profiling, 2D protein electrophoresis, and electron microscopy. Comparative analysis at the transcript and protein level included monocytes and mature endothelial cells as reference cell types.

**Results:**

Our data show that eEPCs and OECs have strikingly different gene expression signatures. Many highly expressed transcripts in eEPCs are haematopoietic specific (RUNX1, WAS, LYN) with links to immunity and inflammation (TLRs, CD14, HLAs), whereas many transcripts involved in vascular development and angiogenesis-related signalling pathways (Tie2, eNOS, Ephrins) are highly expressed in OECs. Comparative analysis with monocytes and mature endothelial cells clusters eEPCs with monocytes, while OECs segment with endothelial cells. Similarly, proteomic analysis revealed that 90% of spots identified by 2-D gel analysis are common between OECs and endothelial cells while eEPCs share 77% with monocytes. In line with the expression pattern of caveolins and cadherins identified by microarray analysis, ultrastructural evaluation highlighted the presence of caveolae and adherens junctions only in OECs.

**Conclusions:**

This study provides evidence that eEPCs are haematopoietic cells with a molecular phenotype linked to monocytes; whereas OECs exhibit commitment to the endothelial lineage. These findings indicate that OECs might be an attractive cell candidate for inducing therapeutic angiogenesis, while eEPC should be used with caution because of their monocytic nature.

## Background

Endothelial Progenitor Cells (EPCs) are a minor population of mononuclear cells circulating in peripheral blood [1]. Although rare in comparison to other blood cells, EPCs are capable of facilitating vascular repair in different ischaemic tissues, therefore they have been regarded as promising candidates for inducing therapeutic angiogenesis in multiple diseases such as acute myocardial infarction, unstable angina, stroke, diabetic microvasculopathies, pulmonary arterial hypertension, atherosclerosis, and ischaemic retinopathies [2-6].

EPCs are classically described as cells expressing a combination of an endothelial marker (VEGFR2) and a progenitor marker (CD34/CD133), however there is considerable debate surrounding this definition, because none of these markers are fully specific [7,8]. It has been reported that CD34+ CD133+ VEGFR2+ cells are haematopoietic and may not actually be true EPCs [9]. Indeed, a methodological comparison of six flow cytometric approaches for EPC quantification using CD34 and VEGFR2 markers has demonstrated only poor to moderate agreement between methods [10].

An alternative approach to isolate EPCs from peripheral or umbilical cord blood utilizes *in vitro *culture and this consistently produces two distinct EPC subtypes which have been named as early EPCs (eEPCs) and Outgrowth endothelial cells (OECs) [11]. OECs are also known as endothelial colony-forming cells (ECFCs)[12] or late EPCs because of their late appearance in culture. Although clear differences have been shown between these two endothelial progenitors, there is still concern surrounding their nature [8], and debate about whether these putative EPCs represent the 'bona fide' EPC [7]. It has been previously demonstrated that both subsets contribute to angiogenesis, but through different mechanisms. eEPCs act in a paracrine manner, while OECs directly incorporate into resident vasculature [13,14]. A range of pre-clinical and clinical studies using EPCs have yielded inconsistent outcomes in terms of therapeutic benefit, implying that a precise EPC definition needs to be elucidated [15]. Moreover, many clinical trials still use very heterogeneous populations of cells such as unfractionated bone marrow or freshly isolated CD34 + cells [2,4]. Clearly, an accurate EPC definition based on a broad range of molecular characteristics is needed. If sub-populations could be precisely characterized, this would facilitate the use of the most appropriate cell therapy in future clinical trials [16]. The aim of this study was to provide a thorough, unbiased analysis of the phenotype of the two EPC subsets isolated *in vitro*; eEPCs and OECs. This was achieved using combined transcriptomic and proteomic analysis to establish a highly detailed molecular fingerprint of these important endothelial progenitors.

## Methods

### Cell Isolation and culture

This study was approved by the Office for Research Ethics Committees Northern Ireland (ORECNI 08/NIR02/20). EPCs were isolated from human peripheral blood (PB) and umbilical cord blood (CB). Fresh human PB (40 mls) was obtained under full ethical approval from three female volunteer subjects aged 25-35, non-smokers, not receiving any medication and without any clinical diagnosis; while CB-derived mononuclear cells (MNCs) were purchased from AllCells LLC (California, USA). MNCs were isolated from PB by density gradient centrifugation. To obtain eEPCs, MNCs were seeded at a density of 2 × 10^6 ^cells/ml onto fibronectin coated petri dishes and cultured in complete EBM-2 MV medium (Lonza Ltd., Slough, UK) that contained hEGF, VEGF, hFGF-B, R^3^-IGF-1 and was supplemented with 10% FBS. OECs were obtained by seeding MNCs onto collagen coated wells at a density of 1 × 10^7 ^cells/ml using complete EBM-2 medium with the same supplements as described above [17]. Monocytes were isolated from peripheral blood by positive selection using CD14 MicroBeads and an autoMACs separator (Miltenyi Biotec, Bergisch Gladbach, Germany). Human dermal microvascular endothelial cells (DMECs) were purchased from PromoCell GmbH (Heidelberg, Germany) and cultured in endothelial cell basal medium MV (Promocell).

### RNA extraction and microarray analysis

Total RNA was extracted using an RNAqueous kit (Ambion, Cambridgeshire, UK). 1 μg of RNA from each cell sample was labelled and hybridised to an Illumina WG-6 v3.0 Expression Beadchip. Samples included in the array analysis were: 3 biological replicates for PB-derived eEPCs and OECs, 3 technical replicates for CB-derived eEPCs and OECs, and single samples for controls DMECs and monocytes. Gene expression data obtained from Illumina Beadstudio was normalised using 'R' bioconductor with 'lumi' package [18]. Data was processed and analysed using the National Institute on Aging (NIA) array, The Database for Annotation, Visualisation and Integrated Discovery (DAVID), GenePattern, and visual anaysis tool (visANT) software.

### Conventional RT-PCR

RT was performed with 500 ng of RNA using random hexamers and Superscript II (Invitrogen, Paisley, UK). Primers were designed using Primer BLAST and tested with AmplifX software. Primer sequences are shown in Additional File 1 Table S1. Conventional RT-PCR was performed in a 30 μl reaction volume containing 1 μl of cDNA, 0.2 μM sense and anti-sense primers designed for the particular gene of interest (Invitrogen), 1× PCR buffer (Invitrogen), 10 mM dNTP mix (Roche, Mannheim, Germany), and 1 μl DNA polymerase (Invitrogen). PCR was performed for 30 cycles using a thermocycler (ABI 2720, Applied Biosystems, Foster City, CA). PCR products were resolved by 2% agarose gel electrophoresis.

### Real time RT-PCR

Quantitative real time RT-PCR reactions were performed in a 10 μl volume containing 2 μl of 1:15 diluted cDNA template, 0.5 μM of sense and anti-sense primers (Invitrogen), and 5 μl of SYBR green mastermix (Qiagen, Crawley, UK). PCR was performed for 45 cycles with denaturation at 94°C for 15 seconds, annealing at 55°C for 30 seconds, and extension at 72°C for 30 seconds using a LightCycler 480 (Roche). Standard curves for quantification of PCR products were constructed using serial dilutions of pooled cDNA.

### Protein Extraction and 2D gel electrophoresis

Protein was extracted by lysing cells in 9 M urea, 2 M thiourea, 4% CHAPS buffer. 7 cm immobilized 4-7 pH gradient (IPG) strips (GE Healthcare Life Sciences, Buckinghamshire, UK) were rehydrated overnight at room temperature in 125 μl of sample containing 100 μg protein. Isoelectric focusing (IEF) was performed on a Pharmacia Biotech Multiphor II tray in a stepwise fashion (Step 1: 500 volts, 1 mA, 5 watts(W) and 5 volt hours (Vhrs); step 2: 3500 volts, 1 mA, 5 W and 5200 Vhrs; and step 3: 3000 volts, 1 mA, 5 W and 3500 Vhrs). After IEF, reduction and alkylation of thiol groups was performed by immersing strips in equilibration buffer containing 10 mg/ml dithiothreitol (DTT) for 15 minutes, and then 27 mg/ml of iodoacetamide (Sigma-Aldrich, UK) for 15 minutes, respectively. Equilibrated strips were placed horizontally on top of NuPAGE 4-12% Bis-Tris gels (Invitrogen) and secured in place by covering it with 0.5% agarose gel (w/v) made up in running buffer and containing trace amounts of bromophenol blue. Electrophoresis was performed starting at 50 V for 30 minutes, then 100 V for up to 2 hours. Gels were fixed for 1 hour in 40% methanol (v/v), 7% acetic acid (v/v) and immersed in Colloidal coomassie solution (Sigma) overnight at room temperature with gentle agitation. Gels were de-stained with 10% acetic acid (v/v), 1% glycerol (v/v) de-stain solution, and scanned using an Odyssey imaging system (Licor Biosciences, UK). Image analysis was performed with Progenesis PG220 software (Nonlinear Dynamics Ltd., Newcastle upon Tyne, UK).

### Electron microscopy

To preserve apico-basal polarity and architecture of cell monolayers, cells were grown on glass coverslips and fixed in 2.5% glutaraldehyde in 0.1 M sodium cacodylate buffer at 4°C overnight. After post-fixation in 1% osmium tetroxide, cells were dehydrated with increasing alcohol concentrations and embedded in Spurr resin. Ultrathin sections for both horizontal and vertical planes were prepared using an ultramicrotome, placed on copper grids (Agar Scientific Ltd, UK), stained with uranyl acetate and lead citrate, and examined using a JEOL 100 CX transmission electron microscope.

## Results

### Human peripheral blood-derived eEPCs and OECs have distinct genomic profiles

**Figure 1:**
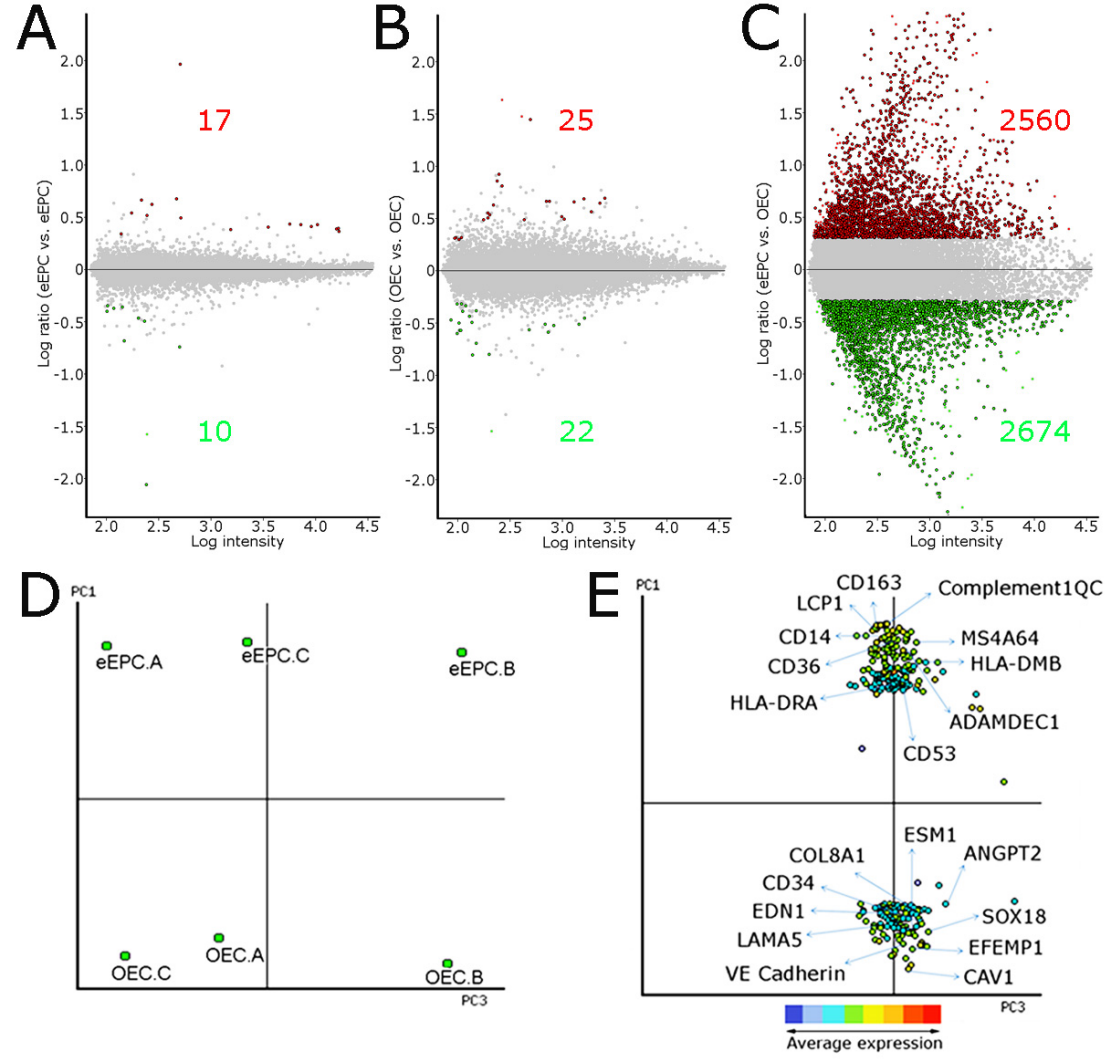
**Peripheral blood-derived eEPC and OECs have distinctive transcriptomes**. Normalised data from Illumina WG-6 v3.0 Expression Beadchip were imported into NIA array for analysis. Numbers of upregulated transcripts are shown in red and downregulated transcripts in green. There were minimal differences (<50 transcripts) among technical replicates as shown by the log-ratio chart for pairwise comparison of eEPC replicates(A), and the log-ratio chart for pairwise comparison of OEC replicates(B); however there were extensive differences (> 5000 differentially expressed transcripts) when eEPCs were compared to OECs as shown by the log-ratio chart for pairwise comparison of eEPCs vs. OECs(C). Prinicipal component analysis (PCA) segregates eEPCs from OECs(D) using one single component (PC1). Biplot analysis on PCA graph identifies transcripts specifically expressed in each EPC subtype(E).

eEPCs and OECs were isolated from human peripheral blood according to established protocols. Each endothelial progenitor type exhibited distinctive and homogeneous cell morphology *in vitro*: eEPCs appeared as spindle-shaped cells (Additional File 1 Figure S1A) that demonstrated a low proliferative potential and no tendency to form contiguous colonies. By contrast, OECs formed confluent cobblestone-shaped monolayers (Additional File 1 Figure S1B) and showed high proliferative potential. Respective immunophenotypes using flow cytometry demonstrated eEPCs express haematopoietic markers CD45 and CD14, while OECs express endothelial markers CD146 and CD105 (R. M. and A. S., manuscript submitted 05/10/09). Total RNA was extracted from 7 day-old eEPC cultures and early passage OECs. Three biological replicates per EPC subtype were used for transcriptome analysis using Illumina WG-6 v3.0 expression beadchips to assay more than 48000 transcripts, including 25,400 well characterised human transcripts and additional confirmed mRNAs in the UniGene database. Paired comparisons using National Institute on Aging (NIA) array analysis determined a correlation greater than 0.98 for eEPC biological replicates, and greater than 0.97 for OEC replicates (Additional File 1 Table S2). However, when comparing eEPCs and OECs, correlation decreased to less than 0.77, demonstrating that these two EPC types differ significantly at the molecular level. Further statistical assessment for genes enriched at least 2 fold with a false discovery rate of 0.01 showed minimal differences (<25 transcripts) between biological replicates belonging to the same EPC subtype (Figure 1A, B), however scatter plots for eEPCs vs. OECs distinguished 2560 transcripts significantly more highly expressed in eEPCs, and 2674 transcripts more highly expressed on OECs (Figure 1C). Lists of the top 20 differentially expressed transcripts in eEPCs/OECs are shown in Table 1 and 2, respectively. The full microarray data is deposited in the NCBI Gene Expression Omnibus (GEO) available at http://www.ncbi.nlm.nih.gov/geo with Accession number GSE20283.

**Table 1:** Identification of differentially expressed transcripts between PB.OECs and eEPCs: Top 20 transcripts higher in eEPCs.

Gene Annotation*	*Symbol*	*Fold Change*
**Complement component 1, q subcomponent, C chain**	**C1QC**	**257.10**
TYRO protein tyrosine kinase binding protein	TYROBP	250.38
**CD163 molecule**	**CD163**	**244.85**
**Secreted phosphoprotein 1**	**SPP1**	**239.94**
**Fc fragment of IgE**	**FCER1G**	**222.58**
**Lymphocyte cytosolic protein 1**	**LCP1**	**220.90**
Glycoprotein nmb, transcript variant B	GPNMB	209.85
**ADAM-like, decysin 1**	**ADAMDEC1**	**206.59**
**Major histocompatibility complex, class II, DR alpha**	**HLA-DRA**	**194.40**
**Membrane-spanning 4-domains, subfamily A, member 6A**	**MS4A6A**	**193.46**
**Major histocompatibility complex, class II, DM alpha**	**HLA-DMA**	**183.40**
Selenoprotein P, plasma 1	SEPP1	181.55
Apolipoprotein E	APOE	176.85
**CD14 molecule**	**CD14**	**176.01**
Matrix metallopeptidase 9	MMP9	173.02
Transmembrane 4L six family member 19	TM4SF19	170.22
**Colony stimulating factor 1 receptor**	**CSF1R**	**164.51**
S100 calcium binding protein A9	S100A9	158.93
Matrix metallopeptidase 7	MMP7	157.78
**Integrin beta 2, lymphocyte antigen 1**	**ITGB2**	**156.86**

*Immune response-related genes highlighted in bold.

**Table 2:** Identification of differentially expressed transcripts between PB.OECs and eEPCs: Top 20 transcripts higher in OECs.

Gene Annotation*	*Symbol*	*Fold Change*
**Caveolin 1**	**CAV1**	**216.27**
EGF-containing fibulin-like extracellular matrix protein 1	EFEMP1	199.8
Procollagen-lysine, transcript variant 2	PLOD2	143.52
Claudin 11	CLDN11	142.59
Four and a half LIM domains 2	FHL2	120.11
**Connective tissue growth factor**	**CTGF**	**106.24**
Guanine nucleotide binding protein	GNG11	106.56
Biglycan	BGN	102.54
**VE-Cadherin**	**CDH5**	**100.55**
Transmembrane 4L six family member 1	TM4SF1	93.95
Matrix metallopeptidase 1	MMP1	89.91
S100 calcium binding protein A16	S100A16	88.25
**Cysteine-rich, angiogenic inducer, 61**	**CYR61**	**88.06**
**Claudin 5**	**CLDN5**	**79.78**
C-type lectin domain family 14, member A	CLEC14A	79.41
Homo sapiens dickkopf homolog 1	DKK1	76.98
Homo sapiens PDZ and LIM domain 1	PDLIM1	71.12
**Endothelial cell-specific molecule 1**	**ESM1**	**69.92**
**SRY (sex determining region Y)-box 18**	**SOX18**	**69.18**
**Laminin, alpha 5**	**LAMA5**	**61.16**

*Endothelial cell -related genes highlighted in bold.

Principal component analysis (PCA) further demonstrated the differences between the mRNA expression profiles of eEPCs and OECs. Using a single component (PC1) it was possible to separate samples into two groups; one containing the three eEPC biological replicates with a high PC1 value, and other group with the three OEC biological replicates showing a low PC1 value (Figure 1D). The PCA biplot analysis highlighted some differentially expressed genes for each group; eEPC genes included HLA-DRA, CD36, CD14, and complement 1QC, while OEC genes comprised caveolin1, VE-cadherin, CD34, and endothelial specific molecule 1, among others (Figure 1E).

**Figure 2:**
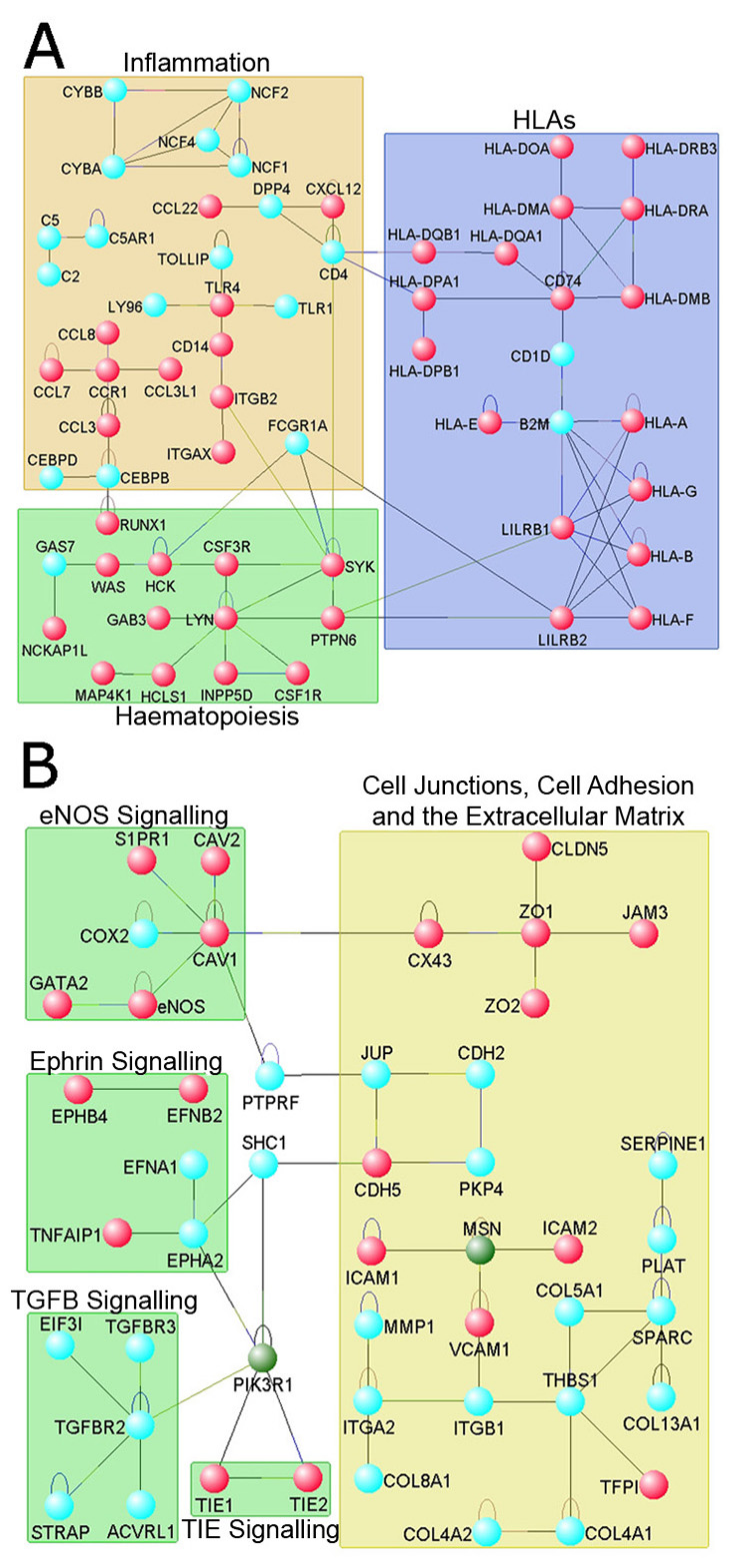
**Transcriptome-based interactome analysis reveals that eEPCs express haematopoietic transcripts and OECs express endothelial transcripts**. Differentially expressed transcripts were imported into visANT software to create gene networks. Diagrams show nodes representing genes, lines indicate interactions between proteins, and boxes denote functional categories. (A) eEPC interactome network, nodes in red are primarily haematopoietic. (B) OEC interactome network, nodes in red are highly expressed in endothelial cells.

In order to gain an insight into the biological meaning behind these long lists of genes, the Database for Annotation, Visualization and Integrated Discovery (DAVID) bioinformatics software was used to assess the top 400 genes differentially expressed in each cell type. The genes preferentially expressed by eEPCs were enriched for those involved in immune response and inflammation, while OECs expressed genes involved in development and angiogenesis (Additional File 1 Table S3). Furthermore we used protein network analysis (interactome) for assessment of genome-wide transcriptomics data. This assumes that the transcriptome profile is a fair, albeit incomplete representation of the proteome. Interactome analysis allows placement of genes identified in microarray experiments in a broader biological context, thus highlighting molecular pathways characteristic of each cell type. Interactome analysis for over 1700 differentially expressed transcripts using the integrative visual analysis tool for biological networks and pathways (visANT) corroborated these findings by showing angiogenesis-related gene networks such as Tie2, eNOS, and Ephrin signalling in OECs (Figure 2A) and inflammation networks including TLRs, HLAs and CYBs in eEPCs. It also demonstrated an abundance of haematopoietic specific elements such as RUNX, WAS, PTPN6, HCLS1 in eEPCs (Figure 2B).

**Figure 3:**
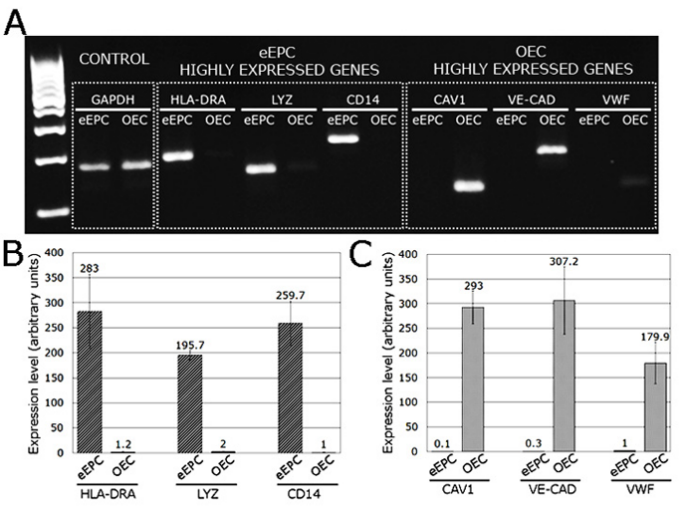
**Expression of characteristic eEPC and OEC genes determined by RT-PCR**. (A) Expression of mRNAs distinctive for eEPCs and OECs determined by semiquantitative PCR: eEPC highly expressed genes HLA-DRA, LYZ, and CD14; OECs highly expressed genes CAV1, VE-CAD, and VWF. (B) Quantitative real time PCR showing gene expression fold changes for the eEPC genes. (C) Quantitative real time PCR showing gene expression fold changes for the OEC genes. Error bars in B and C show mean ± SEM of three biological replicates for each EPC subtype.

To validate gene microarray results, conventional RT-PCR and real time RT-PCR were performed. According to our previous transcript profiling, three highly expressed transcripts for each EPC type were chosen; HLA-DRA, lysozyme, and CD14 for eEPCs, and caveolin1, VE-cadherin, and vWF for OECs. In order to verify that transcriptome analysis could be generalized, eEPCs and OECs from different donors were used for PCR experiments. Conventional RT-PCR at 30 cycles revealed marked higher expression of HLA-DRA, LYZ, and CD14 transcripts in eEPCs, while CAV1, VE-cadherin, and vWF transcripts were only present in OECs (Figure 3A). Similarly, qRT-PCR results were also consistent with microarray results (Figure 3B, C), indicating a good correlation between the two methods, however, as commonly reported greater fold gene expression changes were observed higher (by 1.4-5.6 times) with qRT-PCR than in gene microarray experiments. The microarray and qRT-PCR transcriptome data demonstrate that eEPCs and OECs represent two dissimilar types of endothelial progenitors.

### OECs mRNA fingerprint closely resembles endothelial cells, eEPCs resemble monocytes

Discrepancies exist in the published literature on whether EPCs have monocytic features [19] or not. There is evidence to demonstrate CD14+ cells can generate EPCs that exhibit revascularising properties [20,21]. On the other hand, it has also been suggested that blood monocytes only mimic endothelial progenitors and do not directly contribute to vascular network formation [22]. Furthermore, differentially expressed genes described above (Figures 1 and 2, Tables 1 and 2) suggest monocytic and endothelial characteristics for eEPCs and OECs respectively. Therefore, to elucidate and differentiate the expressed phenotypes of endothelial progenitors, eEPC and OEC transcriptomes were directly compared with monocytes and endothelial cells. Monocytes were isolated from fresh peripheral blood by CD14 positive selection using a magnetic cell sorter and human dermal microvascular endothelial cells (DMECs) were chosen as mature endothelial cells. Total RNA from these two cell types was extracted and used as comparator transcriptomes. CB-derived as opposed to PB-derived eEPCs and OECs were also included in transcriptomic analysis as it has been suggested that foetal blood may represent a richer EPC source than adult peripheral blood [23,24].

**Figure 4:**
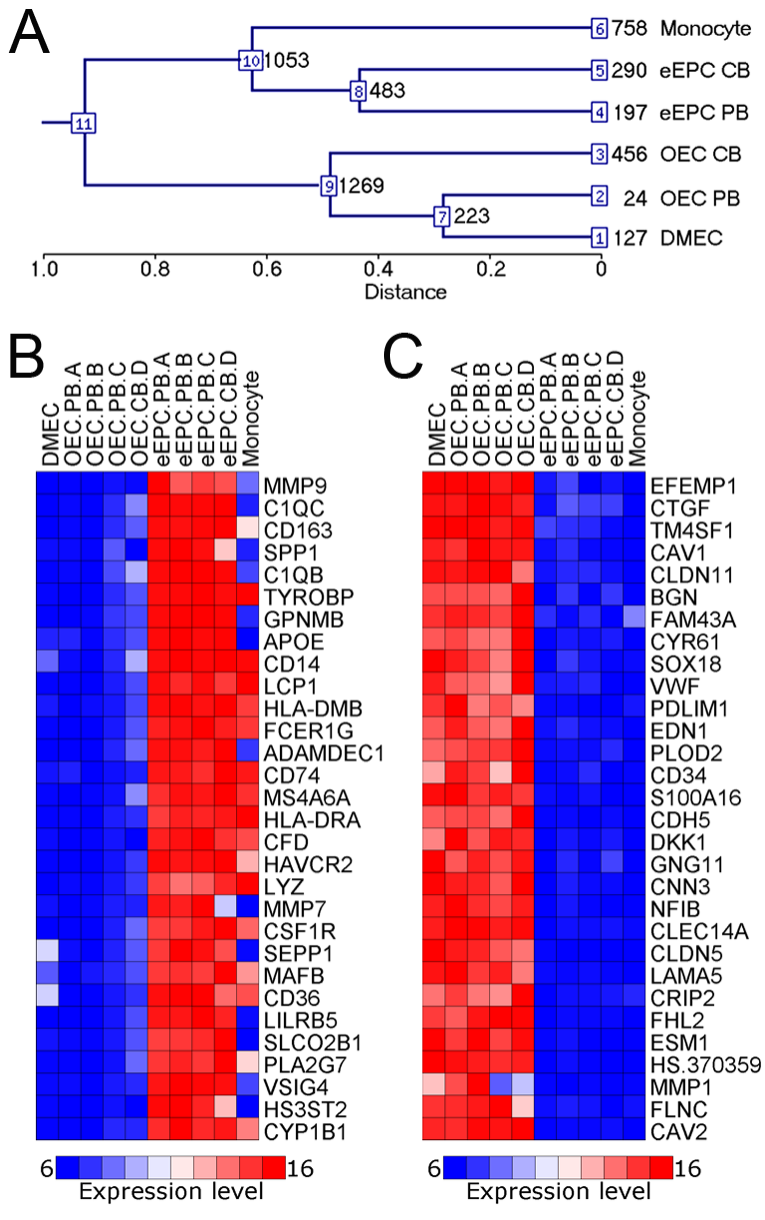
**EPC gene signature relates to monocytes, while OECs are closely linked to endothelial cells**. (A) Hierarchical clustering reveals two major branches: eEPCs are clustered together with monocytes, whilst OECs are grouped with DMECs. Distance represents the similarity of gene expression between different samples (the closer the more similar), and numbers in black indicate statistically significant number of transcripts that identify a cluster; numbers in blue are transcript cluster IDs: clusters 1-6 indicate specific transcripts for a cell type; clusters 7-10 indicate common transcripts for 2/3 cell types; and cluster 11 indicates transcripts present equally in all samples. (B) Heat map of top 30 highly expressed genes in eEPCs demonstrating that eEPCs share a similar gene signature with monocytes. (C) Heat map of top 30 highly expressed genes in OECs indicates a high degree of correlation between OECs and DMECs.

Hierarchical clustering of 21,825 expressed transcripts out of the 48,000 present on the Illumina microarray shows two main branches: one grouping eEPCs with monocytes (1,053 transcripts in common) and the other branch composed of OECs and DMECs (1,269 common transcripts) (Figure 4A). eEPCs from adult PB and foetal CB clustered together and only 483 eEPC-specific transcripts distinguished these cells from monocytes. Interestingly, PB-derived OECs clustered closer to DMECs than to their CB-derived counterparts. Heat maps generated using GenePattern software for the top 30 distinctly expressed transcripts in each EPC subtype show expression patterns at the gene level consistent with hierarchical cluster analysis (Figure 4B, C). As expected, biological replicates of the same EPC type had very similar gene expression profiles. Furthermore, eEPC gene expression patterns partially matched monocytes, and OECs were closely linked to DMECs. Although eEPCs were grouped with monocytes, some subtle mRNA transcription differences were identified such as complement components (C1QC, C1QB), matrix metalloproteinases (MMP9, MMP7, ADAMDEC1) and osteopontin (SPP1) which were higher in eEPCs than in monocytes. This transcriptome analysis of endothelial progenitors, mature endothelial cells and monocytes has added novel information and evidence to support the idea that OECs are progenitors committed to the endothelial lineage; in contrast, eEPCs represent a distinct cell type which resembles monocytes.

### Comparative proteomic analysis of EPCs, monocytes and mature endothelial cells

**Figure 5:**
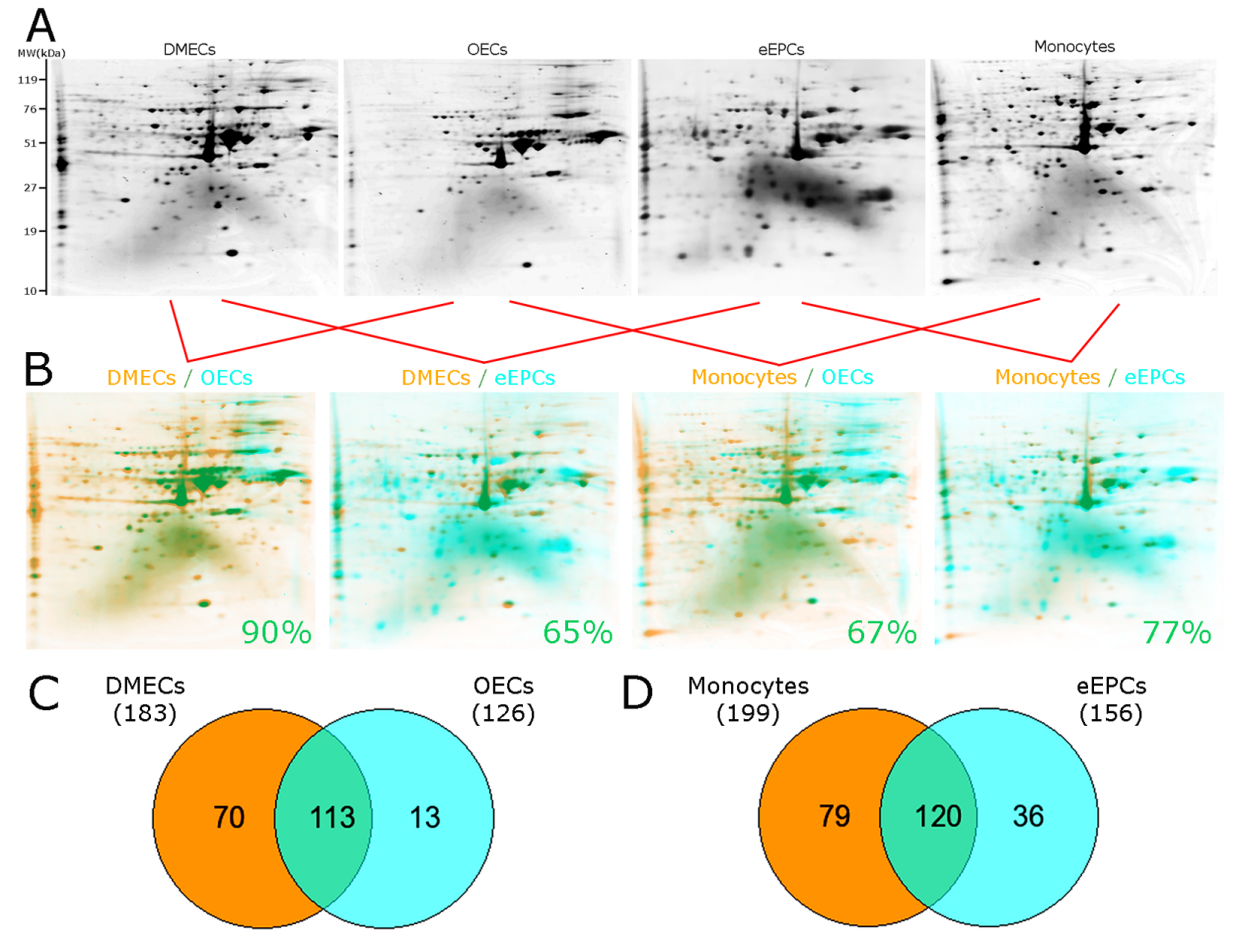
**Comparative proteome analysis of human DMECs, OECs, eEPCs, and monocytes**. (A) Representative images of 2D gels showing protein spots distributed according to their molecular weight vertically; and isoelectric point horizontally from 4 to 7, right to left, respectively. (B) Comparative assessment by overlaying 2D gel images and matching spots. Common spots appear in green colour. Numbers in the bottom right corner show percentage of the progenitor cell proteome that matches the comparator cell type proteome. (C) Venn diagram indicating high overlap between DMECs and OECs proteomes. (D) Venn diagram showing quantification of similar spots between eEPCs and monocytes.

To corroborate the transcriptomics outcomes at the protein level, 2D-PAGE was used to investigate proteomes of each EPC subtype. Protein lysates from monocytes and DMECs were also included to enable comparisons with EPCs. Coomassie blue staining of 2D gels revealed 156 protein spots for eEPCs, 126 for OECs, 183 for DMECs, and 199 for monocytes (Figure 5A). A total of 298 distinct spots were identified in all four groups and 70 spots among them were common to all groups (Additional File 1 Figure S2). For comparative analysis, gels were arbitrarily assigned a different colour (blue or orange), and paired to identify common spots that appeared in a merged colour (green) as shown in Figure 5B. Quantification revealed that eEPCs shared 120 spots with monocytes, indicating 77% of the eEPC protein spot profile is similar to monocytes (Figure 5C). Of note, OECs demonstrated 113 spots in common with DMECs (representative of 90% of the entire protein profile) (Figure 5D and Additional File 1 Table S4). Although we did not characterise protein spots by mass spectrometry, there were 8 spots specific for OECs and 21 spots specific for eEPCs (Additional File 1 Figure S3). These spots might represent important cellular markers for these progenitors. Comparisons by 2D gel overlapping is also possible in a motion picture format that flicks from sample gel to comparator gel allowing easy visual recognition of similarities and differences (Additional Files 2, 3, 4 and 5). These findings demonstrate that the OEC protein profile is closely related to endothelial cells; and the eEPC protein signature is very similar to monocytes.

### OECs have characteristic endothelial ultrastructural features

**Figure 6:**
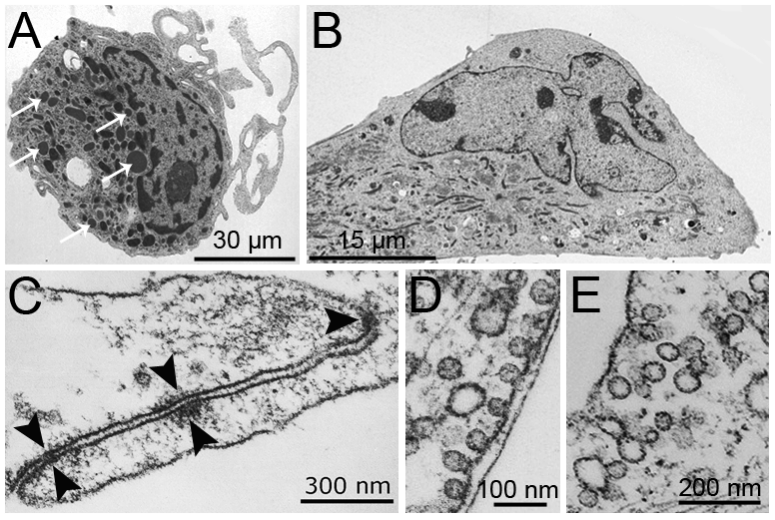
**Ultrastructure analysis identifies typical endothelial features in OECs**. Representative images of eEPCs (A) and OECs (B) by transmission electron microscopy. White arrows indicate lysosome-like structures. Ultrastructural features recognized as adherens junctions indicated by black arrowheads (C), and abundant caveolae (D) in the plasma membrane were only present in OECs. Caveolae display two distinct morphologies, single pits (D) and clusters (E).

Morphological assessment by electron microscopy showed that eEPC (Figure 6A) are very different from OECs (Figure 6B) with only the latter showing apico-basal polarity. OEC microarray data revealed high expression of VE-cadherin, claudin 11, claudin 5, caveolin 1 and caveolin 2 which was indicative of the presence of cell-cell specialized junctions and caveolae organelles. Ultrastructural examination of OECs revealed the presence of adherens junction-like cell-cell contacts identified as discrete areas of electron-dense material on the cytoplasmic face and particles within the intercellular space of 15 nm (Figure 6C). Furthermore, OECs showed abundant flask-like invaginations of the plasma membrane with a diameter of 30-80 nm (Figure 6D). These caveolae occurred both as single pits and as clusters (Figure 6E). Neither caveolae nor adherens junctions were observed on eEPCs, which had multiple lysosome-like structures identified as intracytoplasmic circular electron-dense vesicles of 100-300 nm (Figure 6A). This finding is consistent with the eEPC transcriptome profile that indicated high expression of lysosome enzymes such as lysozyme (LYZ), cathepsins (CTSB, CTSD, CTSH, CTSL1), acid phosphatase (ACP2, ACP5), acid lipase (LIPA), and β-glucoronidase (GUSB). eEPCs also show nuclei containing extensive hetero-chromatin and numerous plasma membrane projections (Figure 6A).

## Discussion

This study has revealed highly distinguishing transcript signatures for eEPCs and OECs and clearly demonstrates that these cells represent distinct EPC populations. We show novel evidence based on transcriptomic-, proteomic-, and ultrastructural- analysis to indicate that eEPCs are haematopoietic cells with a monocytic-like molecular profile, while the OEC molecular fingerprint suggests close association to the endothelial lineage. These findings suggest OECs as candidates to establish cell therapies for ischaemic diseases because of their unmistakable endothelial nature. On the other hand, eEPCs should be used with caution because of their monocytic nature and possible role enhancing tissue inflammation.

In 1997, eEPCs were the first putative endothelial progenitors isolated *in vitro *by culturing CD34+ VEGFR2+ mononuclear blood cells on fibronectin [25] and confirming a subsequent increase in expression of endothelial cell-associated markers such as CD34, CD31, VEGFR2, Tie2, and E-selectin. Later, this protocol was slightly modified by eliminating the cell sorting step and the so-called CFU-Hill colony assay was developed as a commercial kit to quantify EPCs [26]. In recent years, the assertion that CD34+ VEGFR2+ cells are bona fide EPCs has been challenged [9], and the cells growing as CFU-Hill colonies have been genetically linked to primitive haematopoietic cells [12] and suggested to be mainly composed of monocytes and T cells [27]. Furthermore, it has been recently demonstrated that monocytes can acquire endothelial markers *in vitro *by uptake of platelet microparticles, as a simple transfer of antigens CD31 and vWF [28].

The comprehensive transcriptomic data obtained in the current study strengthens the idea that eEPCs are indeed haematopoietic cells with a typical monocytic phenotype. Despite the fact that many studies have shown that eEPCs can promote therapeutic neovascularisation in ischaemic tissues [29], our data suggests that these cells should be utilised for therapy with caution and consideration of the underlying pathology they will treat. This is because the eEPC transcriptome profile indicates a sub-population of cells that are enriched for genes involved in inflammation and immune responses. Therefore, if injected into a pro-inflammatory microenvironment such as in ischaemic diabetic tissues, it is possible they could serve to exacerbate the preexisting pathology [30].

The second putative EPC isolated *in vitro *was firstly named endothelial outgrowth [31], also known as OECs, ECFCs, and late outgrowth endothelial cells. These progenitors were uniformly positive for endothelial markers CD146, thrombomodulin, VEGFR2, VE-cadherin, CD31, CD34, and typically showed greater proliferative potential than circulating endothelial cells. Our group alongside others have established that OECs immunophenotype consists of multiple endothelial markers and complete absence of haematopoietic markers such as CD45 and CD14 [23,32]. The present study adds strong new evidence to prove that OECs possess intrinsic endothelial nature, as revealed in their transcriptome, proteome, and ultrastructure. OECs have been described to be directly involved in vascular repair by forming well-perfused human neo-vessels when injected subcutaneously as matrigel plugs in immune-deficient mice [12,32] and it is important to highlight that the OEC interactome analysis identified essential vasculogenesis/angiogenesis components for cell signalling (Tie2, eNOS, Ephrins, TGFβ) and cell adhesion (adherens, tight and gap junctions, cell adhesion molecules, and collagens). This strongly indicates OECs possess intrinsic angiogenic properties, making them an attractive EPC sub-type to be tested for therapeutic angiogenesis.

Despite exponential growth of EPC literature, the primary origin of OECs is still uncertain. The first report confronting this issue affirms that while circulating endothelial cells are derived from the vessel wall, OECs arise from bone marrow-derived circulating angioblasts [31]. Later, the existence of a 'vasculogenic zone' in the wall of adult human blood vessels that contains EPCs has also been suggested [33], and a complete hierarchy of endothelial progenitor cells including OEC-like cells were isolated from vessel wall-derived cultures [34]. There is even a hypothesis that OECs are 'culture artifacts' arising from *in vitro *conditions [7]. Finding the *in vivo *counterpart for the OECs will resolve this argument, however such a discovery remains challenging due to the rarity of OECs in peripheral blood. Moreover, the lack of a marker combination that uniquely identifies OECs further underscores the timeliness of the current study. The molecular profile for OECs presented in this investigation could ultimately help improve approaches for OECs isolation efficiency from blood or bone marrow.

An important question has emerged around whether adult PB, foetal CB or bone marrow offer the best source for EPC isolation. Many reports recognize the advantages of using CB for greater OEC isolation efficiency in comparison to PB [23], better *ex-vivo *cell number expansion, and even longer stability of vascular networks formed *in vivo *[35,36]. The current study has found that gene expression profile of PB-derived OECs more closely resembles that of mature endothelial cells than those cells derived from CB. Our transcriptomic evaluation also revealed that expression levels of progenitor markers such as CD133, CD34, and C-Kit were higher in CB-derived OECs than in their PB counterparts indicating an apparent cell immaturity which is consistent with their more primitive developmental stage and higher proliferative capacity [24]. By contrast, PB-derived OECs demonstrate higher expression of claudins -11 and -5; which may be indicative of a relative advantage when forming tight junctions with resident endothelium.

## Conclusions

Using genome-wide transcriptional profiling, we have precisely characterized the molecular fingerprint of two distinct EPCs. Furthermore, these progenitor transcriptomes were rigorously compared to mature endothelial cells and monocytes to elucidate on their phenotypic nature, results from this transcriptome assessment were strengthened and confirmed by proteomics and ultrastructure studies. The finding that the molecular fingerprint of OECs corresponds to an endothelial phenotype, while eEPCs are similar to monocytes should inform future studies using these cells to promote revascularization of ischaemic tissues.

Many clinical trials using EPCs have yielded inconsistent outcomes in terms of therapeutic benefit, most of them using very heterogeneous populations of cells. Our results indicate that OECs might be an attractive cell candidate for inducing therapeutic angiogenesis, as their transcript fingerprint, protein profile and ultrastructure clearly indicates they are fully committed to an endothelial phenotype. Our findings also suggest that eEPCs should be used with caution and consideration of the pathology they will treat. If injected into a pro-inflammatory microenvironment, it is possible they could exacerbate pre-existing pathology because of their monocytic nature.

## Competing interests

The authors declare that they have no competing interests.

## Authors' contributions

RM participated in the design of the study; data collection, analysis, and interpretation; and drafted the manuscript. CO participated in the design of the study; data collection, analysis, and interpretation; and drafted the manuscript. MS carried out transcriptomic- and protein profiling- data analysis. JG carried out classical and real time RT-PCR experiments to validate microarray results. TG participated in data analysis and interpretation. DS participated in data analysis and interpretation. AS participated in the design of the study; data analysis and interpretation; and drafted the manuscript. All authors read and approved the final manuscript.

## Pre-publication history

The pre-publication history for this paper can be accessed here:

http://www.biomedcentral.com/1755-8794/3/18/prepub

## Supplementary Material

Additional file 1 Figures S1-S3 and Tables S1-S4 in a word file. Click here for file

Additional file 2 **Video 1**. 2D gel images overlap to compare protein spot profile of eEPCs vs. monocytes. Click here for file

Additional file 3 **Video 2**. 2D gel images overlap to compare protein spot profile of eEPCs vs. mature endothelial cells. Click here for file

Additional file 4 **Video 3**. 2D gel images overlap to compare protein spot profile of OECs vs. mature endothelial cells. Click here for file

Additional file 5 **Video 4**. 2D gel images overlap to compare protein spot profile of OECs vs. monocytes. Click here for file
