# Supplementary material for: Molecular analysis of endothelial progenitor cell (EPC) subtypes reveals two distinct cell populations with different identities
Source: BMC Med Genomics. 2010 May 13;3:18. doi: 10.1186/1755-8794-3-18 (PMC2881111; doi:10.1186/1755-8794-3-18)
Supplement: Additional file 1 — Figures S1-S3 and Tables S1-S4 in a word file. [file 1755-8794-3-18-S1.DOCX]

**ADDITIONAL FILE 1**


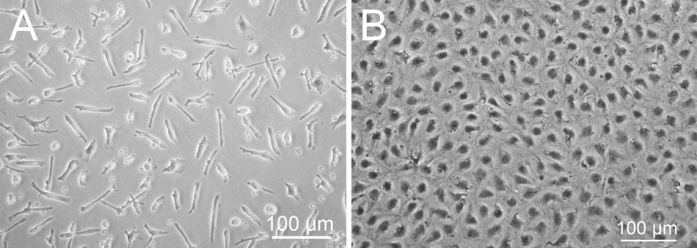

**Figure S1. Distinct morphology of different endothelial progenitors.** (A) eEPCs are spindle-shaped cells. (B) OECs formed cobblestone cell monolayers.


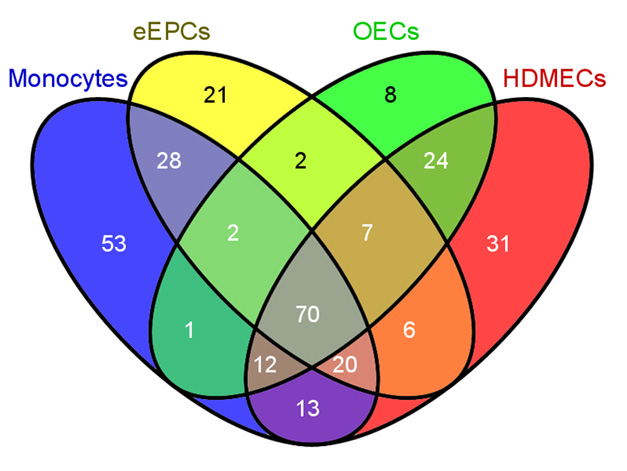


**Figure S2.** Venn diagram of comparative proteomics showing that the highest number of similar protein spots are found in between monocytes/eEPCs and OECs/DMECs.

**
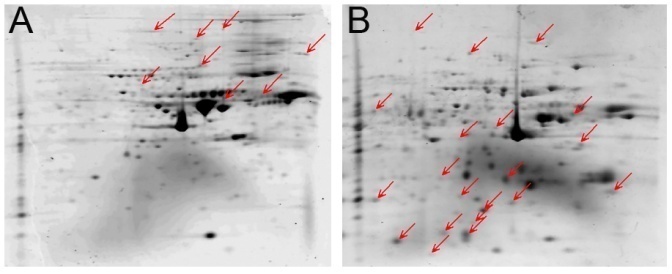
**

**Figure S3.** Protein spots characteristic for OECs (A) and eEPCs (B) are indicated by the red arrows.

**Table S1**

| **Gene** | **Primer sequences** | |
| --- | --- | --- |
| HLA-DRA | Forward | TGGAGTCCCTGTGCTAGGAT |
|  | Reverse | CAGACCGTCTCCTTCTTTGC |
| LYZ | Forward | CTCATTGTTCTGGGGCTTGT |
|  | Reverse | TAACCACTCTCCCATTTGGC |
| CD14 | Forward | AAGAGAGGTGGGGAGGTGAT |
|  | Reverse | CAGCAGCAACAAGCAGGAC |
| CAV1 | Forward | GGGGCAAATACGTAGACTCG |
|  | Reverse | ACTTGCTTCTCGCTCAGCTC |
| VE-CAD | Forward | TGTGGGCTCTCTGTTTGTTG |
|  | Reverse | CGACGATGAAGCTGTATTGC |
| VWF | Forward | TGAGTGCAACGACATCACTG |
|  | Reverse | TGGAGTACATGGCTTTGCTG |

**Table S2**

| **Correlation Matrix* for Log-intensity values of PB eEPCs vs. OECs** | | | | | | |
| --- | --- | --- | --- | --- | --- | --- |
|  | 1 | 2 | 3 | 4 | 5 | 6 |
| 1. eEPC.PB.A | 1 | 0.983 | 0.989 | **0.769** | **0.764** | **0.767** |
| 2. eEPC.PB.B | 0.983 | 1 | 0.988 | **0.774** | **0.769** | **0.768** |
| 3. eEPC.PB.C | 0.989 | 0.988 | 1 | **0.769** | **0.766** | **0.770** |
| 4. OEC.PB.A | **0.769** | **0.774** | **0.769** | 1 | 0.972 | 0.967 |
| 5. OEC.PB.B | **0.764** | **0.769** | **0.766** | 0.972 | 1 | 0.986 |
| 6. OEC.PB.C | **0.767** | **0.768** | **0.770** | 0.967 | 0.986 | 1 |
| ** Values generated by paired comparison of samples using NIA array.* | | | | | | |

**Table S3**

| **Clusters of differentially expressed transcripts** | | | |
| --- | --- | --- | --- |
| **eEPC transcripts** | | **OEC transcripts** | |
| **Annotation cluster** | **Enrichment score*** | **Annotation cluster** | **Enrichment score*** |
| Immune response | 9.30 | Developmental process | 2.81 |
| Inflammatory response | 9.25 | Vasculature development | 2.81 |
| Response to stress | 9.25 | Angiogenesis | 2.81 |
| ** Value obtained by assessing enrichment of functional categories using DAVID bioinformatics software.* | | | |

**Table S4**

| **Percentage of protein spots that were similar to cell comparator proteome profile** | | | | | |
| --- | --- | --- | --- | --- | --- |
|  | | **OECs** | **DMECs** | **eEPCs** | **Monocytes** |
| **OECs** | *Percentage* | **100%** | 90% | 64% | 67% |
|  | *Number of spots* | **126** | 113 | 81 | 85 |
| **DMECs** | *Percentage* | 62% | **100%** | 56% | 62% |
|  | *Number of spots* | 113 | **183** | 102 | 113 |
| **eEPCs** | *Percentage* | 52% | 65% | **100%** | 77% |
|  | *Number of spots* | 81 | 102 | **156** | 120 |
| **Monocytes** | *Percentage* | 43% | 57% | 60% | **100%** |
|  | *Number of spots* | 85 | 113 | 120 | **199** |
